# Effects of chronic crocin treatment on desoxycorticosterone acetate (doca)-salt hypertensive rats

**Published:** 2014-01

**Authors:** Mohsen Imenshahidi, Bibi Marjan Razavi, Ayyoob Faal, Ali Gholampoor, Seyed Mehran Mousavi, Hossein Hosseinzadeh

**Affiliations:** 1 Pharmaceutical Research Center, Department of Pharmacodynamy and Toxicology, School of Pharmacy, Mashhad University of Medical Sciences, Mashhad, Iran; 2 Targeted Drug Delivery Research Center, Department of Pharmacodynamy and Toxicology, School of Pharmacy, Mashhad University of Medical Sciences, Mashhad, Iran; 3 School of Pharmacy, Mashhad University of Medical Sciences, Mashhad, Iran; 4 Pharmaceutical Research Center, Department of Pharmacodynamy and Toxicology, School of Pharmacy, Mashhad University of Medical Sciences, Mashhad, Iran

**Keywords:** Crocin, *Crocus sativus*, Saffron, Systolic blood pressure, Tail cuff

## Abstract

***Objective(s):*** In this study, the effects of chronic administration of crocin, an active constituent of saffron, on blood pressures of normotensive and desoxycorticosterone acetate (DOCA) - salt induced hypertensive rats, were investigated.

***Materials and Methods:*** Five week administration of three doses of crocin (50, 100 and 200 mg/kg/day) and spironolactone (50 mg/kg/day) in different groups of normotensive and hypertensive rats (at the end of 4 weeks treatment by DOCA-salt) was carried out and their effects on mean systolic blood pressure (MSBP) and heart rate (HR) were evaluated using tail cuff method. The duration of effect of crocin on SBP, was also evaluated.

***Results:*** Our results indicated that chronic administration of crocin could reduce the MSBP in DOCA salt treated rats in a dose dependent manner. Crocin did not decrease the MSBP in normotensive rats. The data also showed that antihypertensive effects of crocin did not persist.

***Conclusion:*** It is concluded that crocin possesses antihypertensive and normalizing effect on BP in chronic administration.

## Introduction

*Crocus sativus* L. (saffron), is a perennial stem-less herb which belongs to the Iridaceae family. It is widely cultivated in Iran and other countries (1). Crocin is a carotenoid isolated from *C. sativus* and is responsible for the red color of saffron. It is conside-red a pharmacologically active component of saffron. Modern pharmacological studies have demonstrated that crocin can be used as a new therapeutic agent. It has antitumor (2, 3), antioxidant, radical scavenging (4), hypolipidemic (5), antinociceptive and antiinfla-mmatory (6, 7), anticonvulsant (8), antidepressant (9), and memory-improving effects (10, 11). Crocin also showed protective effects on diazinon and acrylamide induced oxidative stress both in *in vitro* and *in vivo* experiments (12-14). Cardiovascular effects of saffron and its components have been established in some studies. A potent inhibitory effect of aqueous-ethanol extract of *C. sativus* on heart rate and contractility of guinea pig heart via calcium channel-blocking effect, has been shown (15). Furthermore it was indicated that *C. sativus* petal extract possesses hypotensive effect in rats (16). The hypotensive effects of saffron stigma aqueous extract as well as two major constitutes of this plant, crocin and safranal, in normotensive and hypertensive anaesthetized rats have been shown in our previous study (17). Although the effect of this plant in lowering blood pressure has been shown previously, effect of crocin on blood pressure in chronic administration has not been studied. Thus, in this study the effects of chronic administration of crocin on blood pressures of normotensive and desoxycorticosterone acetate (DOCA)-salt induced hypertensive rats were investigated. 

## Material and Methods

***Animal and chemicals***

Adult male Wistar rats (weight 250–300 g) were provided by animal center (School of Pharmacy, Mashhad University of Medical Sciences). They were maintained on a 12 hr light/dark cycle and at a temperature of 23±1^°^C with free access to food and water. These conditions were kept constant through-out the experiments. All animal experiments were carried out in accordance with Ethical Committee Acts of Mashhad University of Medical Sciences. Crocin was dissolved in saline (0.9% NaC1); saline (0.9% NaC1) was used as a negative control.

***Plant and extracts***

*C. sativus* L. stigma were collected from Ghaen (Khorasan province, Northeast Iran) and analyzed in accordance with ISO/TS 3632-2. Crocin was extract-ed and purified as defined by Hadizadeh and colleagues (18). Briefly, saffron stigma powder was suspended in ethanol 80% at 0°C. After centrifuga-tion, the supernatant was separated. Then 80% ethanol was added to the sediment and the extracti-on was repeated; this step was repeated six more times. For preparation of crocin, the resulting solute-on was kept in a thick-walled glass container at -5ºC for 24 days in darkness. The container was sealed during this period. The obtained crystals were separated from the solution and washed with acetone to remove the remaining water. The total amount of crocin in the saffron extract was determined as 10–15%. 

***Induction of experimental hypertension***

Hypertension was induced using desoxycort-icosterone acetate (DOCA)-salt (20 mg/kg, twice weekly, for 4 weeks, SC) and NaCl (1%) in rat’s drinking water (17). Rats were randomly divided into 7 groups; 1) Saline injected (0.5 ml/kg, twice weekly, SC, for 4 weeks), this treatment was conti-nued for another five weeks, 2) (DOCA)-salt (20 mg/kg, twice weekly, for 4 weeks, SC), DOCA treatment was followed by IP injection of 0.5 ml/kg normal saline for another five weeks, 3, 4 and 5) (DOCA)-salt (20 mg/kg, twice weekly, for 4 weeks, SC), DOCA treatment was followed by IP injection of 50, 100 and 200 mg/kg/day crocin for another five weeks, after that crocin injection was stopped but DOCA injection was continued for another two weeks, 6) (DOCA)-salt (20 mg/kg, twice weekly, for 4 weeks, SC), DOCA treatment was followed by IP injection of 50 mg/kg/day spironolactone for another five weeks, after that spironolactone inject-tion was stopped but DOCA injection was continued for another two weeks, 7) Saline injected (0.5 ml/kg, twice weekly, SC, for 4 weeks), saline treatment was followed by IP injection of 200 mg/kg/day crocin for another five weeks. All groups consisted of six rats.

**Figure 1:**
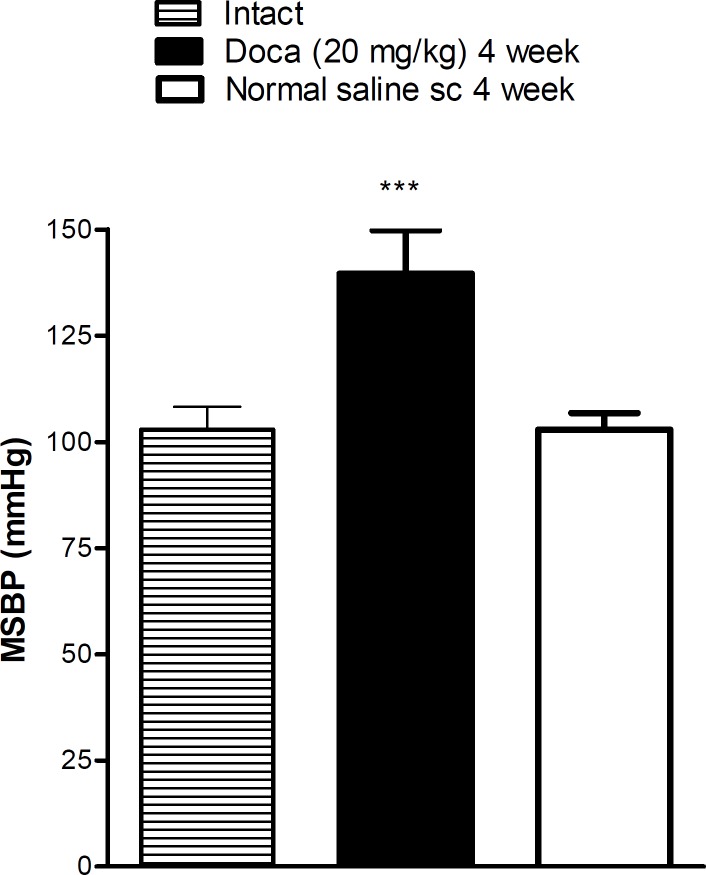
Hypertension induced by Desoxycorticosterone acetate (DOCA)-salt after 4 weeks. Each value is the mean± SEM of six experiments, *** *P*<0.001 vs normal saline treated rats. One way ANOVA, Tukey Krumer test

***Hypotensive activity***

Four, nine, and eleven weeks after the ﬁrst saline or DOCA treatment, SBP was measured using tail cuff method in all groups as described by Lorenz (19). Briefly, three days before the last treatment, the training of rats in different groups for indirect SBP measurements was started. Training consisted of regular handling of the animals and getting them used to the restraining cage and the tail-cuff. Rats were heated for approximately 15 min at 30–32°C to increase blood flow to the tail. After that, animals were placed in small restraining cages with a cuff around the end of proximal of the tail. After placing the cuff, a pulse transducer was used around the end of the tail. Then the tail cuff was inflated using the related button on the NIBP (Non-Invasive Blood Pressure) controller apparatus and data acqui-sition was performed by Power Lab (ADInstruments, v 5.4.2) computerized system. The mean values of five BP and HR readings were used for each animal.

***Statistical analysis***

All data are presented as mean ± SEM. The statistical comparisons among groups in each experi-ment were done with one-way analysis of variance (ANOVA) followed by Tukey-Kramer test for multiple comparison. *P*-values less than 0.05 were considered significant. 

## Results

***Effect of DOCA on SBP***

 In DOCA treated rats, MSBP significantly increased in comparison with normal saline treated (normotensive) rats (*P*<0.001) (Figure 1).

***Effects of crocin in normotensive and hypertensive rats after nine weeks***

**Figure 2:**
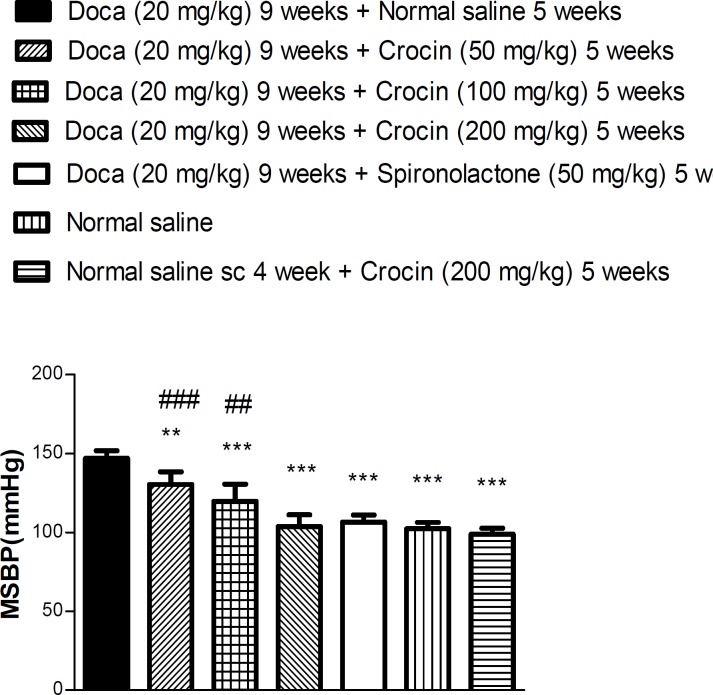
Mean systolic blood pressure (MSBP) in response to various doses of crocin in normotensive and hypertensive rats at the end of nine weeks. Each value is the mean ± SEM of six experiments. One-way ANOVA, Tukey Krumer,^**^P<0.01 and ***P<0.001vs DOCA plus normal saline treated rats, ^##^*P*<0.01 and ^###^*P*<0.001 vs DOCA plus spironolactone

The injection of crocin (50, 100 and 200 mg/kg) decreased the MSBP in hypertensive animals (*P*<0.0 1 and *P*<0.001, respectively) (Figure 2), but in norm-otensive rats, crocin did not reduce the MSBP. The hypotensive effect of crocin was dose dependent; in the highest dose, it was similar to that of spironol-actone.

**Figure 3:**
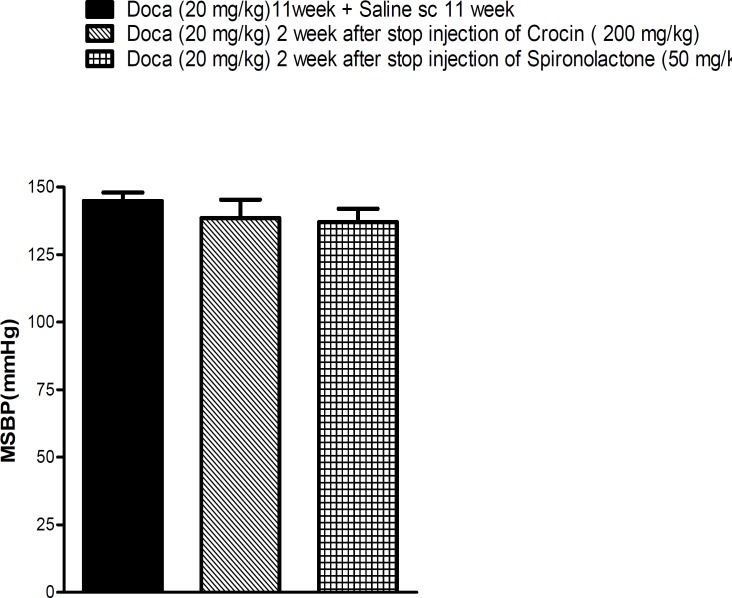
Evaluation of the duration hypertensive effect of crocin. Each value is the mean ± SEM of six experiments. One-way ANOVA, Tukey Krumer

***Evaluation of duration effect of*** ***crocin on SBP***

 As shown in Figure 3, the decreasing level of SBP at the highest doses of crocin as well as spironol- actone did not persist in rats, and after stopping the administration at the end of eleven weeks, SBP increased again. 

## Discussion

Deoxycorticosterone acetate (DOCA)- salt is an agent commonly used to induce hypertension in experimental animals (17). Our results showed that DOCA-salt significantly induced hypertension in comparison with saline group at the end of 4 weeks of treatment. Crocin reduced the increase of MSBP induced by DOCA in chronic exposure, dose dependently, but this hypotensive effect was not observed in normotensive rats. 

Vasodilatory effects of saffron and its constituents have been proved in previous studies. For example, potent relaxant effect of *C. sativus* and safranal on smooth muscles of guinea pigs has been shown (20). Furthermore crocin could inhibit the extracellular Ca^2+^ inﬂux and release of intracellular Ca^2+^ in endopl-asmic reticulum in cultured bovine aortic smooth muscle cells (21). As a result of the reduction of intracellular Ca^2+^release and blood vessel relaxation, hypotension could occur (22), so it might be concluded that hypotensive effect of crocin in chronic treatment is related to the blocking of calcium channel or inhibition of sarcoplasmic reticu-lum Ca^2+^ release into cytosol. Also, it was indicated that aqueous and ethanolic extracts of saffron petals, reduced the mean arterial blood pressure in anaesth-etized rats (16). Moreover it was revealed that aqueous extract of saffron stigma and two major components of this plant have hypotensive effects in normotensive and hypertensive anaesthetized rats via intravenous injection (17). Similar to the results of our previous study, crocin did not cause reﬂex tachycardia (data was not shown), so it could be concluded that both heart function and blood vessel contractility may be affected by crocin (17). 

Based on pathophysiological and biochemical changes following administration of DOCA-salt in rats, it is believed that DOCA-salt hypertensive rats, provide an animal model of oxidative and inflame-matory stress in the cardiovascular system (23). Hence, the DOCA-salt experiment can provide an appropriate model for evaluation of anti-oxidative or anti-inflammatory effects of natural or synthetic compounds on cardiovascular system. This also provides opportunities for the development of novel therapeutic agents for management of chronic cardiovascular disease (24). Therefore, it could be concluded that the antihypertensive effects of crocin could be related partly due to its antioxidant properties (4). It is well known that DOCA induced hypertension causes an endothelial dysfunction in the isolated aortic rings as well as in the perfused mesenteric bed (24). As crocin decreased SBP in hypertensive rats, our results may show that the vasodilatory effect of crocin was endothelium depen-dent. It was also shown that crocetin, another active component of saffron, inhibited down regulation of expression of eNOS as a result of oxidized LDL and increased NO production in BAECs (25). Hence, the hypotensive effect of crocin could be partly due to the increased activity of eNOS, resulting in increase of NO production and vasodilatation and improve-ment of DOCA impaired endothelial dependent relax-ation. 

According to our results, chronic administration of crocin did not reduce MSBP in normotensive rats, this observation was supported by our previous study which showed that crocin alone did not have hypotensive effect on SBP but improved the toxic effects of diazinon on blood pressure in concurrent administration (12).

Administration of deoxycorticosterone acetate (DOCA) plus high salt intake (DOCA-salt hyperten-sion) in rats has been extensively studied as an experimental animal model of mineralocorticoid-dependent hypertension. Increased blood volume and increased blood pressure are due to increased DOCA-induced reabsorption of salt and water (26). Spironolactone, known as a potassium-sparing diur-etic, inhibits the effects of aldosterone by comp-eting for intracellular mineralocorticoid receptors in the cortical collecting duct. This decreases the reabs-orption of sodium and water, and the secretion of potassium (27). Hence, in this study spironolactone was used as a positive control. Our results showed that the antihypertensive effect of crocin at the highest dose was as much as spironolactone at the end of nine weeks. It is likely that the hypotensive effect of crocin may be due to the saffron diuretic effects (1, 28). 

To evaluate the duration of reducing effects of crocin on SBP, the injection of crocin was stopped at the end of nine weeks, but DOCA injections were continued for another two weeks. The data showed that antihypertensive effects of crocin did not persist, so it could be postulated that long term blood pressure regulation systems were not affected by crocin. 

## Conclusion

In summary, our results indicated that in chronic administration, crocin could reduce the MSBP in DOCA salt treated rats, in a dose dependent manner. 

## References

[1] Ríos J, Recio M, Giner R, Manez S (1996). An update review of saffron and its active constituents. Phytother Res.

[2] Fernández J (2006). Anticancer properties of saffron, Crocus sativus Linn. Adv Phytomed.

[3] Hosseinzadeh H, Behravan J, Ramezani M, Ajgan Kh (2005). Anti-tumor and cytotoxic evaluation of Crocus sativus L. stigma and petal extracts using brine shrimp and potato disc assays. J Med Plants.

[4] Hosseinzadeh H, Shamsaie F, Mehri S (2010). Antioxidant activity of aqueous and ethanolic extracts of Crocus sativus L. stigma and its bioactive constituents crocin and safranal. Pharmacogn Mag.

[5] Sheng L, Qian Z, Zheng S, Xi L (2006). Mechanism of hypolipidemic effect of crocin in rats: crocin inhibits pancreatic lipase. Eur J Pharmacol.

[6] Amin B, Hosseinzadeh H (2012). Evaluation of aqueous and ethanolic extracts of saffron, Crocus sativus L., and its constituents, safranal and crocin in allodynia and hyperalgesia induced by chronic constriction injury model of neuropathic pain in rats. Fitoterapia.

[7] Vosooghi S, Mahmoudabady M, Neamati A, Aghababa H (2012). Crocin alleviates the local paw edema induced by histamine in rats. Avicenna J Phytomed.

[8] Hosseinzadeh H, Talebzadeh F (2005). Anticonvulsant evaluation of safranal and crocin from Crocus sativus in mice. Fitoterapia.

[9] Hosseinzadeh H, Karimi G, Niapoor M (2004). Antidepressant effects of Crocus sativus stigma extracts and its constituents, crocin and safranal, in mice. J Med Plants.

[10] Hosseinzadeh H, Sadeghnia HR, Ghaeni FA, Motamedshariaty VS, Mohajeri SA (2012). Effects of saffron (Crocus sativus L.) and its active constituent, crocin, on recognition and spatial memory after chronic cerebral hypoperfusion in rats. Phytother Res.

[11] Hosseinzadeh H, Ziaei T (2006). Effects of Crocus sativus stigma extract and its constituents, crocin and safranal, on intact memory and scopolamine-induced learning deficits in rats performing the Morris water maze task. J Med Plants.

[12] Razavi M, Hosseinzadeh H, Abnous K, Motamedshariaty V, Imenshahidi M (2013). Crocin restores hypotensive effect of subchronic administration of diazinon in rats. Iran J Basic Med Sci.

[13] Hariri A, Moallem S, Mahmoudi M, Hosseinzadeh H (2011). The effect of crocin and safranal, constituents of saffron, against subacute effect of diazinon on hematological and genotoxicity indices in rats. Phytomedicine.

[14] Mehri S, Abnous K, Mousavi S, Motamed Shariaty V, Hosseinzadeh H (2012). Neuroprotective effect of crocin on acrylamide-induced cytotoxicity in PC12 cells. Cell Mol Neurobiol.

[15] Boskabady MH, Shafei MN, Shakiba A, Sang Sefidi H (2008). Effect of aqueous-ethanol extract from Crocus sativus (saffron) on guinea-pig isolated heart. Phytother Res.

[16] Fatehi M, Rashidabady T, Hassanabad ZF (2003). Effects of petals extracts of saffron on rat blood pressure and on responses induced by electrical field stimulation in the rat isolated vas deferens and guinea–pigileum. J Ethnopharmacol.

[17] Imenshahidi M, Hosseinzadeh H, Javadpour Y (2010). Hypotensive effect of aqueous saffron extract (Crocus sativus L.) and its constituents, safranal and crocin, in normotensive and hypertensive rats. Phytother Res.

[18] Hadizadeh F, Mohajeri S, Seifi M (2010). Extraction and purification of crocin from saffron stigmas employing a simple and efficient crystallization method. Pak J Biol Sci.

[19] Lorenz JN (2002). A practical guide to evaluating cardiovascular, renal, and pulmonary function in mice. Am J Physiol Regul Integr Comp Physiol.

[20] Boskabadi MH, Aslani MR (2006). Relaxant effect of crocus sativus (saffron) on guinea pig tracheal chains and its possible mechanisms. J Pharm Pharmacol.

[21] He S, Qian Z, Tang F (2004). Effect of crocin on intracellular calcium concentration in cultured bovine aortic smooth muscle cells. Acta Pharm Sin.

[22] Williams B, Liu C, Deyoung L, Brock G, Sims S (2005). Regulation of intracellular Ca2+ release in corpus cavernosum smooth muscle: synergism between nitric oxide and cGMP. Am J Physiol Cell Physiol.

[23] Lyer A, Chan V, Brown L (2010). The DOCA-Salt hypertensive rat as a model of cardiovascular oxidative and inflammatory stress. Curr Cardiol Rev.

[24] Fatehi-Hassanabad Z, Fatehi M, Imen Shahidi M (2004). Endothelial dysfunction in aortic rings and mesenteric beds isolated from deoxycorticosterone acetate hypertensive rats: possible involvement of protein kinase C. Eur J Pharmacol.

[25] Iwazu Y, Muto S, Fujisawa G, Nakazawa E, Okada K, Ishibashi S (2008). Spironolactone suppresses peritubular capillary loss and prevents deoxycorticosterone acetate/salt-induced tubulointerstitial fibrosis. Hypertension.

[26] Tang FT, Qian ZY, Liu PQ, Zheng SG, He SY, Bao LP (2006). Crocetin improves endothelium-dependent relaxation of thoracic aorta in hypercholesterolemic rabbit by increasing eNOS activity. Biochem Pharmacol.

[27] Cheng S, Suzuki K, Sadee W, Harding B (1974). Effects of spironolactone, canrenone and canrenoate-K on cytochrome P450, and 11beta- and 18-hydroxylation in bovine and human adrenal cortical mitochondria. Endocrinology.

[28] Mousavi SZ, Bathaie SZ (2011). Historical uses of saffron: Identifying potential new avenues for modern Research. Avicenna J Phytomed.

